# Genome-wide identification and functional characterization of the *KWL* gene family in three *Oryza* species

**DOI:** 10.3389/fpls.2025.1707474

**Published:** 2025-11-19

**Authors:** Lyu Wu, Wei Liu, Guimin Xin, Yang Chen, Haitao Zhou

**Affiliations:** 1 Northeast Agricultural Research Center of China, Jilin Academy of Agricultural Sciences, Gongzhuling, China; 2 Institute of Biotechnology Research, Chongqing Academy of Agricultural Sciences, Chongqing, China

**Keywords:** KWL, genome-wide identification, evolutionary relationships, phytohormone response, rice

## Abstract

**Introduction:**

The Kiwellin (KWL) gene family, although previously implicated in plant stress responses, remains poorly characterized in rice. This study aims to perform a comprehensive genome-wide analysis of the KWL family across three rice species to elucidate their roles.

**Methods:**

We analyzed the KWL family in Oryza sativa ssp. japonica (Os), O. sativa ssp. indica (Osi), and O. rufipogon (Or). Systematic analyses of their phylogeny, gene structures, conserved motifs, chromosomal localization, and promoter cis-elements were performed. The expression patterns of key genes in response to phytohormones were validated, and their subcellular localization and transcriptional activity were experimentally determined.

**Results:**

A total of 33 KWL genes were identified (9 in Os, 12 in Osi, and 12 in Or). Phylogenetic analysis revealed that KWL proteins were highly conserved within rice species but distinct from their maize and tomato orthologs. Promoter cis-element analysis revealed a significant enrichment of elements associated with biotic/abiotic stress responses and phytohormone signaling. Expression profiling demonstrated that most family members exhibited low or tissue-specific expression, with OsKWL1 and OsKWL2 exhibiting marked responsiveness to ABA and JA treatments in roots and shoots, respectively. Subcellular localization experiments confirmed that both OsKWL1 and OsKWL2 proteins were localized to the cytoplasm. Furthermore, yeast assays revealed that OsKWL1 possessed strong autoactivation activity.

**Conclusion:**

This study establishes a foundational framework for future functional investigations of the KWL family in rice, highlighting its potential roles in stress adaptation.

## 1 Introduction

Kiwellin (KWL) proteins were first discovered in kiwifruit. Rich in cysteine, they function as allergenic proteins capable of triggering allergic reactions in humans (Tamburrini et al., 2005; Tuppo et al., 2008; Wang et al., 2019). Previous studies revealed that Kiwellin proteins are widely distributed across most plant species, including monocotyledons, Asteraceae, and Rosaceae. However, they have not been identified in Brassicaceae plants such as *Arabidopsis thaliana*. Additionally, Kiwellin coding sequences have been detected in the genomes of certain fungi that infect plants (Han et al., 2019).

Extensive research supported the role of Kiwellin as a plant defense molecule, with numerous studies demonstrating that its encoding gene was significantly upregulated under diverse biotic stresses in multiple plant species. For instance, transcripts of two Kiwellin homologs in potato (*Solanum tuberosum*) were found to be significantly upregulated following infection by the pathogenic *Phytophthora infestans* (Draffehn et al., 2013; Mosquera et al., 2016). Similarly, the Kiwellin gene *RRP1* in pepper (*Capsicum annuum*) was strongly induced following infection with Pepper golden mosaic virus (PepGMV), reaching its expression peak at the early stage of disease development (6 dpi), and declining during the subsequent recovery phase (20 dpi) (Gongora-Castillo et al., 2012). Furthermore, transcriptomic data indicate that two *KWL* genes (*SIKWL2* and *SIKWL3*) in tomato (*Solanum lycopersicum*) were also strongly induced after infection with *Phytophthora infestans*. Notably, their induction was significantly stronger in resistant cultivars than in susceptible ones, suggesting their potential involvement in plant disease resistance (Su et al., 2024).

Although numerous studies have demonstrated that *KWL* genes are significantly induced under biotic stress, their precise physiological functions and underlying molecular mechanisms in plants remain to be thoroughly elucidated. Currently, functional investigations have only been reported in maize and cotton. In maize, two paralogous proteins, ZmKWL1 and ZmKWL1-b, constitute a redundant defense system that preserves the integrity of the salicylic acid (SA) signaling pathway by specifically inhibiting the enzymatic activity of the virulence effector UmCmu1 secreted by *Ustilago maydis*, thereby enhancing resistance to smut disease (Altegoer et al., 2020; Djamei et al., 2011; Han et al., 2019). In cotton, GhKWL1 mediates resistance to Verticillium wilt by upregulating the transcriptional activity of *GhERF105*. However, this defense mechanism can be compromised by the pathogenic effector VdISC1, secreted by *Verticillium dahliae*, through its direct interaction with GhKWL1 (Chen et al., 2021; Liu et al., 2014).

Rice, a staple food for over half of the world’s population, faces significant yield losses due to various environmental stresses (Fan et al., 2023; Zhu, 2016). In response to these challenges, plants employ sophisticated signaling networks orchestrated by hormones (Verma et al., 2016; Waadt et al., 2022). For instance, abscisic acid (ABA) is pivotal in abiotic stress responses (Li et al., 2025; Liu et al., 2022, 2023; Xie et al., 2024), while salicylic acid (SA), jasmonic acid (JA), and ethylene (ET) are central to defense against biotic stresses (Li et al., 2013; Ma et al., 2020; Meng et al., 2020). However, despite the well-established role of these hormonal pathways, the function of specific gene families in mediating these responses in rice remains largely unexplored. Notably, the *KWL* gene family has not been systematically identified or characterized in rice. Given the significant genomic differences among *Oryza* species that have arisen during evolution, this study aims to fill this knowledge gap (Wang et al., 2018). We performed a genome-wide analysis of the *KWL* family in *Oryza sativa* ssp. *japonica*, *Oryza sativa* ssp. *indica* and *Oryza rufipogon*. Furthermore, we investigated the expression profiles of *OsKWL* genes in response to key plant hormones. Our findings establish a crucial foundation for future functional studies of *KWL* genes in rice stress biology.

## 2 Materials and methods

### 2.1 Identification of *KWL* genes in three *Oryza* species

The genome and annotation files of *Oryza sativa* ssp. *japonica* (Nipponbare, abbreviated as *Os*) were obtained from the online database (http://www.ricesuperpir.com/) (Shang et al., 2023). The genome and annotation files of *Oryza sativa* ssp. *indica* (Minghui 63, abbreviated as *Osi*) were retrieved from the RIGW database (http://rice.hzau.edu.cn/rice_rs3/), while those of common wild rice (*Oryza rufipogon*, abbreviated as *Or*) were downloaded from the Ensembl Plants database (https://plants.ensembl.org/). Initially, the previously characterized KWL protein sequences from maize were used as queries for BLAST searches to obtain preliminary candidates of the gene family (Han et al., 2019). Subsequently, the hidden Markov model (PF24300) corresponding to the conserved domain of the *KWL* gene family was downloaded from the InterPro database (https://www.ebi.ac.uk/interpro/). Based on this model, the TBtools software was used to search for homologous proteins across the whole-genome protein datasets (Chen et al., 2023). Finally, the NCBI CDD Search tool (https://www.ncbi.nlm.nih.gov/Structure/cdd/wrpsb.cgi) was used for further validation to ensure that each putative member contained the kiwellin domain. The physicochemical properties of the identified KWL proteins, including amino acid length, molecular weight (MW), and theoretical isoelectric point (pI), were analyzed using the Protein Parameter Calc module of TBtools. Subcellular localization prediction was performed using DeepLoc-2.1 (https://services.healthtech.dtu.dk/services/DeepLoc-2.1/).

### 2.2 Construction of the phylogenetic tree

Protein sequences of the *KWL* gene family members in maize and tomato were obtained from the MaizeGDB (https://maizegdb.org/) and Sol Genomics database (https://solgenomics.net/), respectively. Multiple sequence alignment of the KWL proteins from the three *Oryza* species, maize, and tomato was performed using the ClustalW program implemented in MEGA X software (Kumar et al., 2018). A phylogenetic tree was subsequently constructed using the Neighbor-Joining (NJ) method with 1000 bootstrap replicates. The resulting tree was visualized and annotated using the online tool iTOL (https://itol.embl.de/).

### 2.3 Gene structure and motif analysis

We used TBtools to visualize the gene structures of *KWL* family members based on the genome annotation files of the three *Oryza* species. Conserved motifs were predicted using the MEME suite (https://meme-suite.org/meme/tools/meme), with the maximum number of motifs set to 10 and all other parameters set to default. The results of these analyses were subsequently visualized with TBtools.

### 2.4 Protein tertiary structure prediction

Protein tertiary structures were predicted using the AlphaFold3 platform (Jumper et al., 2021). The model with the highest confidence score (model_0) was selected for further analysis. Three-dimensional structural visualization was performed with PyMOL.

### 2.5 Chromosomal localization and collinearity analysis

The chromosomal locations of the *KWL* genes were determined based on the genome and annotation files of the three *Oryza* species using TBtools. Gene duplication events within the *KWL* family were analyzed using the MCScanX algorithm implemented in TBtools. For comparative collinearity analysis, *Oryza sativa* ssp. *japonica* (Nipponbare) was selected as the reference species, and collinear relationships between its *KWL* genes and those in maize, sorghum, tomato, and kiwifruit were examined. The KaKs Calculator tool in TBtools was subsequently used to calculate Ka, Ks, and Ka/Ks ratios of the duplicated gene pairs.

### 2.6 Analysis of cis-acting elements in promoters

The 2.0 kb promoter sequences upstream of the transcription start sites of all *KWL* genes were extracted using TBtools. The extracted sequences were submitted to the PlantCARE online database (https://bioinformatics.psb.ugent.be/webtools/plantcare/html/) for identification of cis-acting elements. The results were visualized using TBtools.

### 2.7 Gene expression analysis

Expression data of *OsKWLs* across various tissues, including root, seed, panicle, leaf, and shoot, were retrieved from the Rice Expression Database (Project DRP000391; https://ngdc.cncb.ac.cn/red/index). The data were organized and used to generate expression heatmaps via the HeatMap module in TBtools. Additionally, transcriptome data of *OsKWL* genes under abscisic acid (ABA) and jasmonic acid (JA) treatments were obtained from the Rice RNA-seq Database (https://plantrnadb.com/ricerna/), and corresponding expression heatmaps were also generated with TBtools.

### 2.8 Plant materials, growth conditions, and hormone treatments

The *japonica* rice cultivar Nipponbare was used in this study. Rice plants were hydroponically grown in a controlled growth chamber under a 14 hr light/10 hr dark photoperiod, with a constant temperature of 30°C and a light intensity of 66%. After two weeks of growth, uniform seedlings were transferred to fresh nutrient solutions containing either 100 μM ABA or 100 μM JA. Seedlings grown in hormone-free nutrient solution were used as controls. Shoot and root samples were harvested at 1, 3, 6, 12, and 24 hr after treatment, immediately frozen in liquid nitrogen, and stored at –80°C for RNA extraction.

### 2.9 qRT-PCR analysis

Total RNA was extracted using the TIANGEN RNAprep Pure Kit (TIANGEN Technology Company, Beijing, China) following the manufacturer’s protocol, which includes an on-column DNase I digestion step to remove genomic DNA contamination. The integrity and purity of RNA were assessed by agarose gel electrophoresis and spectrophotometric analysis (A260/A280 ratio). Then, 1 μg of purified RNA was reverse-transcribed into cDNA using the FastKing One Step RT-PCR Kit to ensure complete removal of any residual genomic DNA. Quantitative PCR (qRT-PCR) was performed following the instructions of the FastFire qPCR SYBR Green Master Mix (TIANGEN Technology Company, Beijing, China). *OsActin* was used as an internal reference gene and three biological replications were performed. Relative expression levels of target genes were calculated using the 2^−ΔΔCT^ method. The primer used for qRT-PCR are listed in **Supplementary Table S3**.

### 2.10 Subcellular localization

Rice protoplasts were isolated from the etiolated shoots of two-week-old Nipponbare seedlings. Briefly, leaf tissues were finely sliced and digested in an enzyme solution containing 1.5% cellulose R10 and 0.75% macerozyme R10 dissolved in 0.6 M mannitol solution for 6 hr in the dark with gentle shaking. The released protoplasts were purified by filtration through a nylon mesh and collected by centrifugation.

For transfection, the coding sequences without the stop codon of *OsKWL1* and *OsKWL2* were inserted into the pAN580 vector such that it was driven by the CaMV 35S promoter. Approximately 10 μg of purified OsKWL1-GFP or OsKWL2-GFP plasmid DNA was then mixed with 200 μL of protoplast suspension and an equal volume of PEG solution. The mixture was incubated at room temperature for 20 minutes. Subsequently, the transfection was stopped by adding W5 solution, and the protoplasts were collected by centrifugation. The transfected protoplasts were resuspended in WI solution and incubated in the dark at 28°C for 15 hr before confocal microscopy observation, as previously described with modifications (Ma et al., 2017). GFP fluorescence was detected with a confocal scanning laser microscope (LSM800, Zeiss, Jena, Germany). To further observe subcellular localization, the p35S::OsKWL1-GFP and p35S::OsKWL2-GFP vectors were separately expressed in *Nicotiana benthamiana* leaf epidermal cells via *Agrobacterium tumefaciens*-mediated transformation. Primers used in these assays are listed in **Supplementary Table S3**.

### 2.11 Transcriptional activation activity analysis

The Y2H system was used to verify self-activation activity. The CDS of *OsKWL1* and *OsKWL2* were cloned into the pGBKT7 vector and co-transformed with the pGADT7 vector into the Y2HGold yeast (*Saccharomyces cerevisiae*) strain. The transformed yeast cells were plated on SD/-Leu-Trp medium and incubated upside down in a 30°C incubator for approximately 3 d. The combinations pGBKT7-53+pGADT7-T and pGBKT7+ pGADT7-T were used as positive and negative controls, respectively. Single colonies were subsequently selected and transferred onto SD/-Leu-Trp/-His/-Ade medium for further cultivation and observation. The primers used are listed in **Supplementary Table S3**.

## 3 Results

### 3.1 Identification and characterization of *KWL* genes in three *Oryza* species

To identify KWL proteins in three *Oryza* species, we performed homologous alignment using maize KWL protein sequences, followed by further validation with the NCBI-CDD and Pfam databases. Ultimately, 9, 12, 12 members were identified to be KWL genes in *Os*, *Osi*, and *Or* rice species, respectively (**Supplementary Table S1**). These members were designated as *OsKWL1* to *OsKWL9*, *OsiKWL1* to *OsiKWL12*, and *OrKWL1* to *OrKWL12* based on their chromosomal positions. Further analysis revealed significant variations in the physicochemical properties among these members. The resulting amino acid lengths ranged from 74 amino acids (OrKWL8) to 1202 amino acids (OsKWL7), and the protein molecular weights varied from 7893.80 Da (OrKWL8) to 139015.14 Da (OsKWL7). Based on prediction results, the isoelectric points (pI) of these family members ranged from 4.36 (OsKWL2/OrKWL3) to 10.47 (OsiKWL10). Among them, 12 members exhibited pI values greater than 7, suggesting their classification as basic proteins, while the remaining 21 members had pI values below 7, indicating acidic proteins. The instability index ranged from 37.09 (OsiKWL8) to 67.08 (OsKWL6). Among these, only OsiKWL8 exhibited an instability index below the threshold of 40, indicating its potential as a stable protein. OrKWL5 had the highest aliphatic index (84.33), while OsKWL2 had the lowest aliphatic index (65.57). Furthermore, except for OsiKWL4, the GRAVY (Grand Average of Hydropathicity) values of all other members were below zero, indicating that, aside from OsiKWL4, the remaining members were hydrophilic proteins, with OsKWL7 exhibiting the strongest hydrophilicity. Subcellular localization predictions indicated predominant targeting to the nucleus, cell membrane, or chloroplast.

### 3.2 Phylogenetic analysis

**Figure 1:**
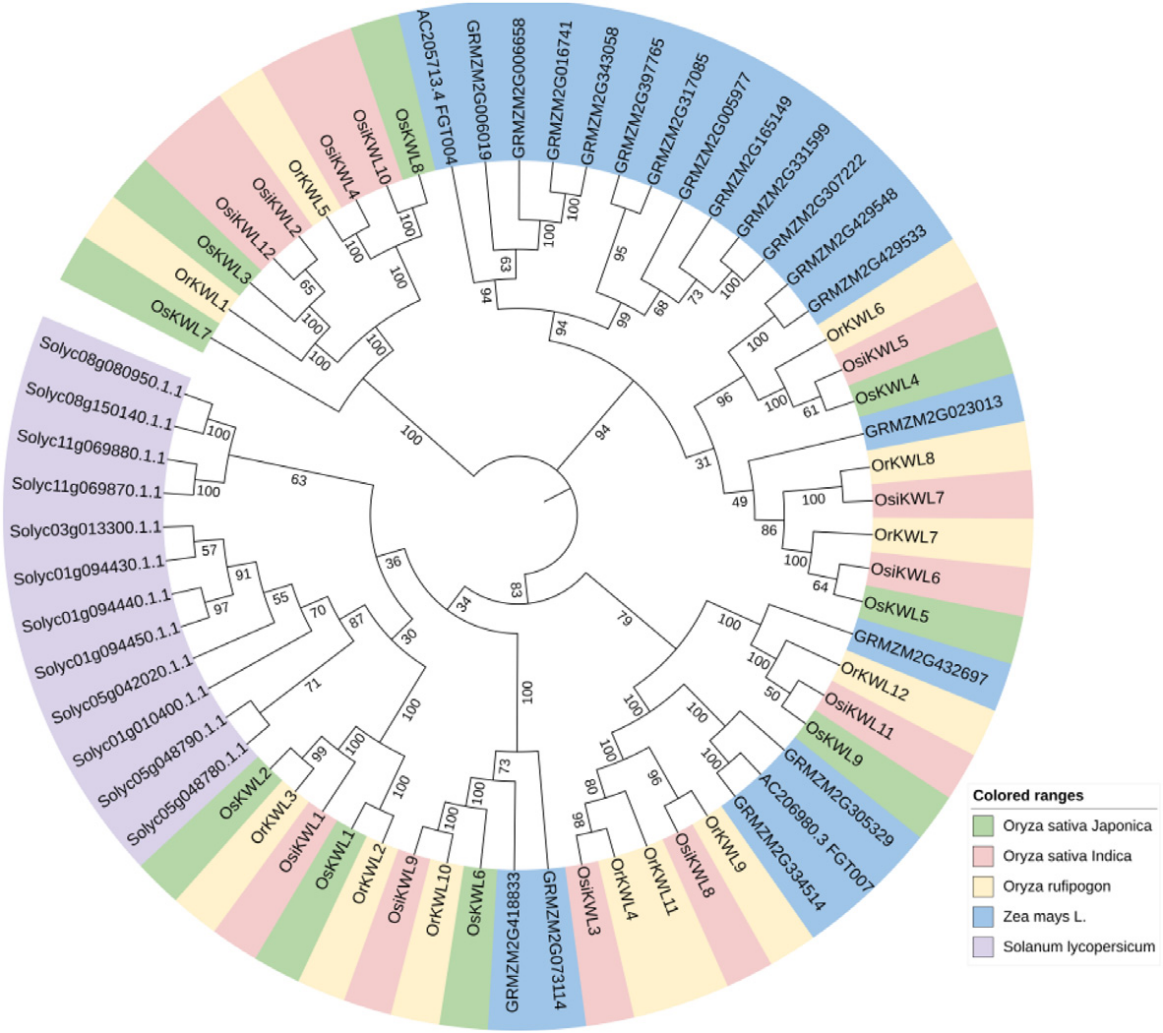
Phylogenetic tree of *KWL* members from three rice species, *Solanum lycopersicum* and *Zea mays*, constructed using the neighbor-joining method (1000 bootstrap replicates). In the phylogenetic tree, homologous members across species are color-coded uniformly.

To elucidate the evolutionary relationships among KWL proteins across three *Oryza* species, we constructed a phylogenetic tree using the neighbor-joining method, including members of the *KWL* gene family from maize (*Zea mays*) and tomato (*Solanum lycopersicum*) (**Figure 1**). The results revealed a high degree of conservation among *KWL* members from the three rice species.

### 3.3 Gene structures and conserved motifs analysis

**Figure 2:**
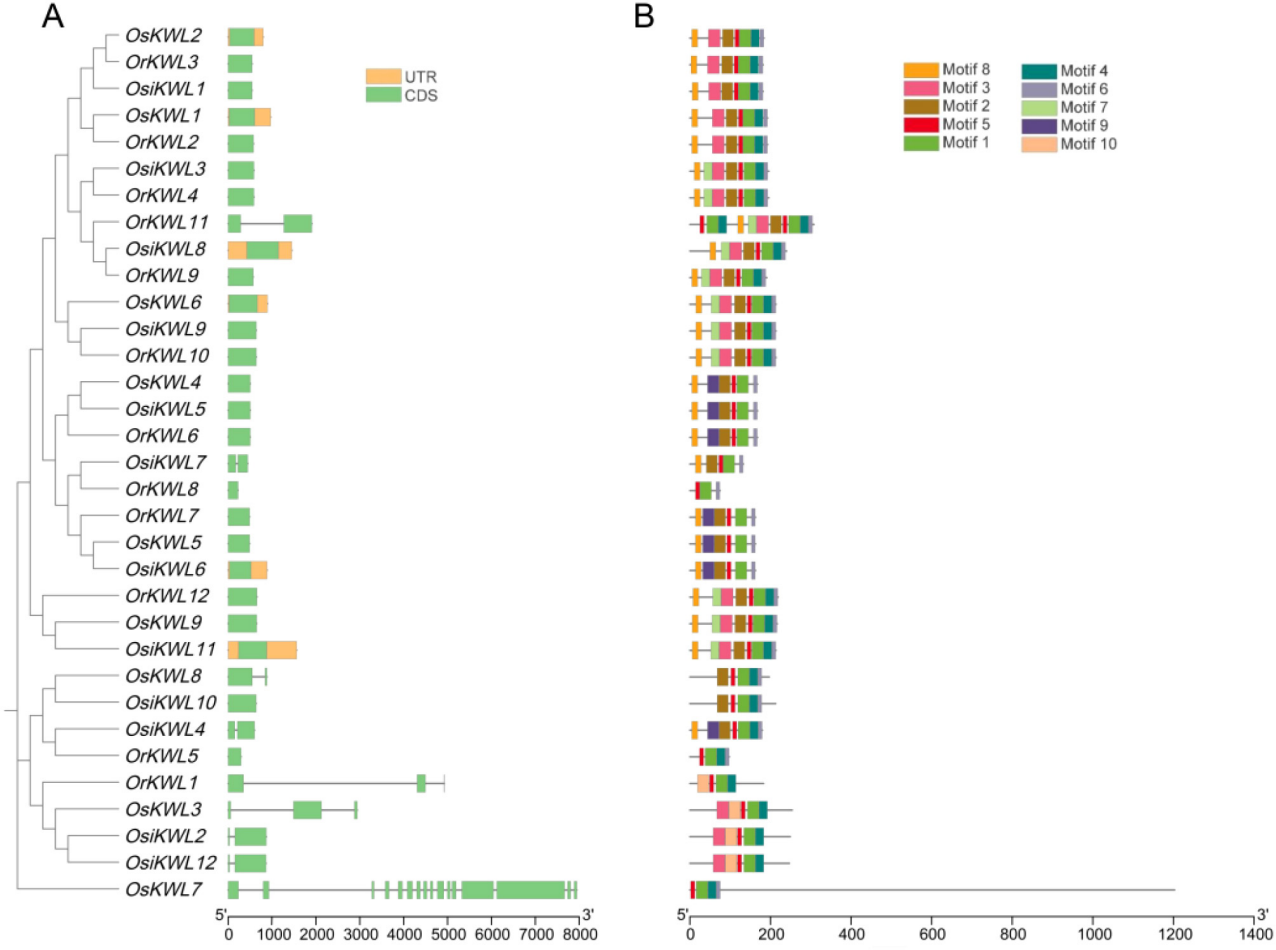
The gene structure and conserved protein motif of *KWLs* in three rice species. **(A)** Evolutionary analysis of *KWLs*, and gene structure visualization of *KWLs* in *Os*, *Osi*, and *Or*. **(B)** conserved motif visualization.

To investigate the structural evolution of *KWL* genes, we conducted a comparative analysis of gene structures among the three *Oryza* species based on the phylogenetic tree (**Figure 2A**). The most prominent feature was the prevalence of intronless genes (single exon), which was conserved in the majority of members across all three subspecies, suggesting a strong evolutionary constraint favoring a compact gene structure. Beyond this conserved pattern, both shared and lineage-specific structural variations were detected. For instance, the unique multi-exonic structure of *OsKWL7* (16 exons) had no structural orthologs in either *Osi* or *Or*, indicating a potential *japonica*-specific innovation. In contrast, genes with 2 or 3 exons (e.g., *OsKWL3/8*, *OsiKWL2/4/7/12*, *OrKWL1/11*) were present in all subspecies. However, these genes did not always form clear orthologous groups, implying independent evolutionary trajectories or divergent evolution following duplication.

To further elucidate the structural diversity and potential functional divergence of *KWL* genes, we next examined the distribution and conservation of conserved motifs among the three *Oryza* species (**Figure 2B**). A total of ten conserved motifs were identified, and their distribution largely mirrored the phylogenetic topology. Core motifs, such as Motif1 and Motif5, were universally present in all proteins from *Os*, *Osi*, and *Or*, suggesting that they constitute an essential functional core of the *KWL* family. Lineage-specific motifs were also observed; for instance, Motif10 was exclusively detected in four members (OrKWL1, OsKWL3, OsiKWL2, and OsiKWL12) that clustered within the same phylogenetic clade. Notably, proteins belonging to the same orthologous group, as defined by our synteny analysis, consistently shared an identical motif composition. This strong correspondence highlights the functional conservation of these orthologous groups during the evolution of *Oryza* species. In contrast, OrKWL8, with only three motifs, may represent a divergent or degenerative form within the *KWL* family.

Together, these findings suggest that the structural simplicity and conserved motif composition of *KWL* genes have been largely maintained throughout rice evolution, while occasional lineage-specific modifications may have contributed to their functional diversification.

### 3.4 Prediction of the tertiary structure of KWL proteins

The prediction of protein tertiary structures can provide valuable insights into the evolutionary relationships among gene family members. Using the AlphaFold3 platform, we successfully predicted the tertiary structures of KWL proteins, revealing that OsKWL7 displays the most complex structural architecture (**Supplementary Figure S2**). Notably, KWL proteins within the same phylogenetic clade demonstrate higher structural similarity, as exemplified by the comparable models of OsKWL2/OrKWL3 and OsKWL9/OsiKWL11. These findings establish a crucial foundation for further comprehensive investigations into the functional networks of *KWL* gene family members.

### 3.5 Chromosomal localization and gene duplication analysis

**Figure 3:**
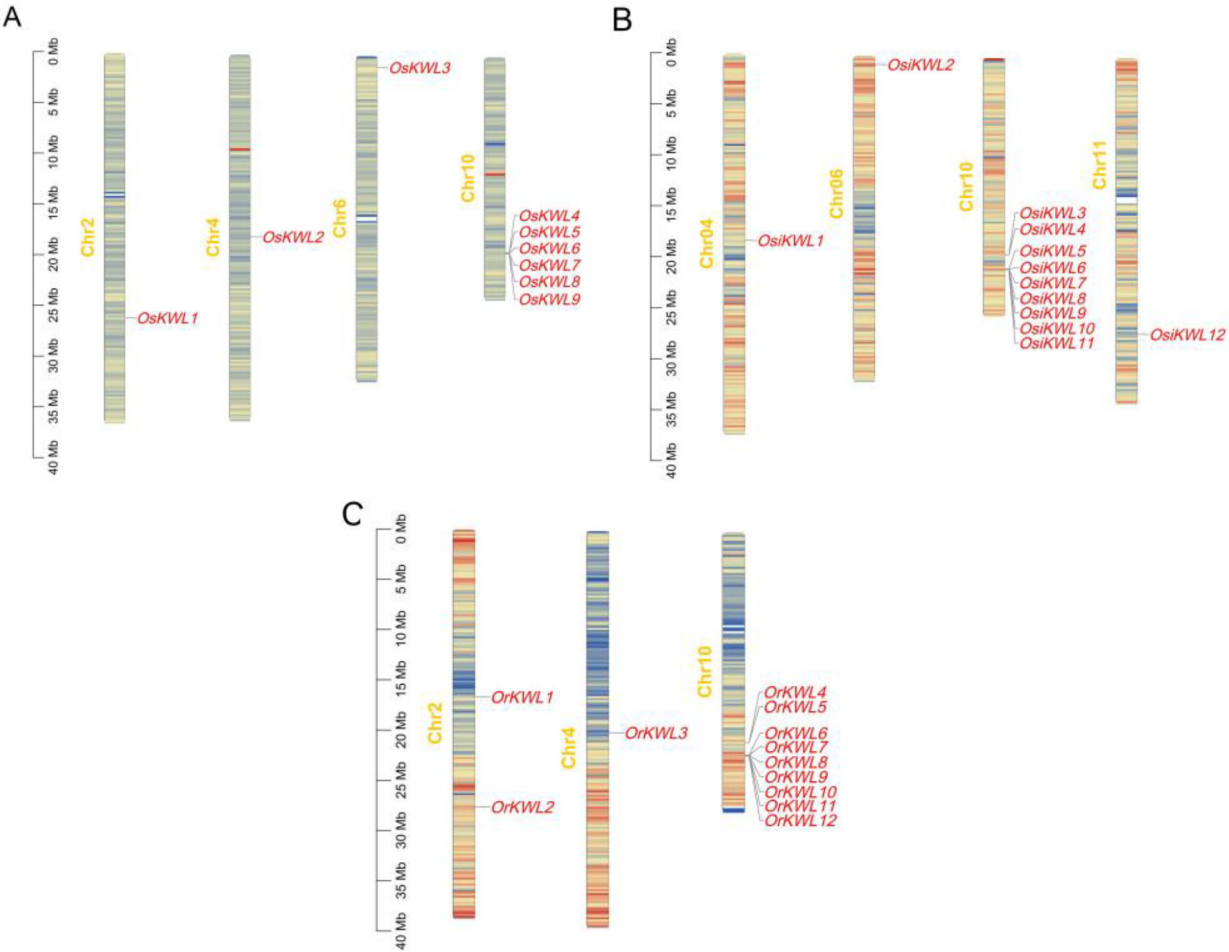
Chromosomal distribution of *KWL* genes in the three rice species. **(A)***Oryza sativa* ssp. *japonica*. **(B)***Oryza sativa* ssp. *indica*. **(C)***Oryza rufipogon*. Gene density per chromosome was calculated using a sliding window of 200 kb. The color gradient from blue to red represents low to high gene density, respectively.

A comparative analysis of chromosomal distribution revealed both a conserved hotspot and subspecies-specific patterns of *KWL* gene localization (**Figure 3**). A striking commonality across all three *Oryza* subspecies was the predominant clustering of *KWL* genes on chromosome 10, which harbored 6, 9, and 9 genes in *Os*, *Osi*, and *Or*, respectively. Beyond this conserved cluster, genomic distribution varied among the subspecies: *Os* possessed additional genes on chromosomes 2, 4, and 6; *Osi* on chromosomes 4, 6, and 11; and *Or* on chromosomes 2 and 4. Given that gene family expansion is typically driven by duplication events, we performed a systematic synteny analysis to investigate the evolutionary mechanisms underlying this distribution. Interestingly, no collinear gene pairs were detected among the three subspecies (**Supplementary Figure S3**). This absence of recent segmental or tandem duplications suggests that the *KWL* family did not expand independently within each lineage after divergence. Instead, the observed gene number differences (9 in *Os vs*. 12 in *Osi*/*Or*) may be attributed to lineage-specific gene loss or to ancient duplication events followed by rapid sequence divergence, which may have erased detectable syntenic relationships.

### 3.6 Comparative collinearity analysis

**Figure 4:**
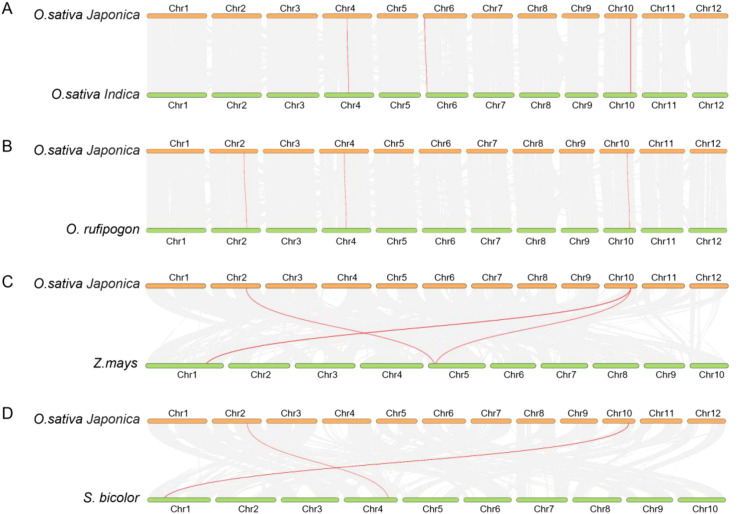
Synteny analysis of *KWL* genes among *O. sativa Japonica, O. sativa Indica, O. rufipogon, Z. mays*, and *S. bicolor*. **(A)** O. sativa Japonica vs. O. sativa Indica. **(B)** O. sativa Japonica vs. O. rufipogon. **(C)** O. sativa Japonica vs. Z. mays. **(D)** O. sativa Japonica vs. S. bicolor. Each small short line represents a chromosome. The gray lines represent all collinear gene pairs between genomes, while the red lines indicate collinear relationships specifically between KWL genes across species.

We selected *Os* as the primary species and identified homologous genes in *Osi*, *Or*, monocots (maize [*Zea mays*] and sorghum [*Sorghum bicolor*]), and dicots (tomato [*Solanum lycopersicum*] and kiwifruit [*Actinidia deliciosa*]). The analysis revealed that *Os* and *Osi* shared four homologous gene pairs, while three genes in *Os* showed collinearity with three genes in *Or* (**Figures 4A, B**). Notably, chromosomal distributions of *KWL* genes exhibited high synteny between *Os* and the other two rice species, suggesting low divergence of *KWL* genes during evolution. Furthermore, *Os* shared four homologous gene pairs with monocots (maize and sorghum) (**Figures 4C, D**), but no significant collinearity was detected with dicots (tomato and kiwifruit) (**Supplementary Figure S4**), indicating closer evolutionary relationships between rice *KWLs* and monocots and suggesting potential core biological functions of *KWL* genes in monocots.

To further explore the selective pressures acting on *KWL* genes during evolution, we calculated the nonsynonymous substitution rate (Ka), synonymous substitution rate (Ks), and Ka/Ks ratios for these homologous gene pairs (**Supplementary Table S2**). Results showed that Ka and Ks values were both 0 in four homologous pairs, indicating no accumulation of synonymous or nonsynonymous mutations. Additionally, the *OsKWL2*/*OsiKWL1* pair exhibited a Ka/Ks ratio >1 (potential positive selection), while the remaining 10 pairs had Ka/Ks ratios <1, demonstrating that rice *KWL* genes are predominantly under purifying selection during evolution.

### 3.7 Cis-acting element analysis of *KWL* genes

**Figure 5:**
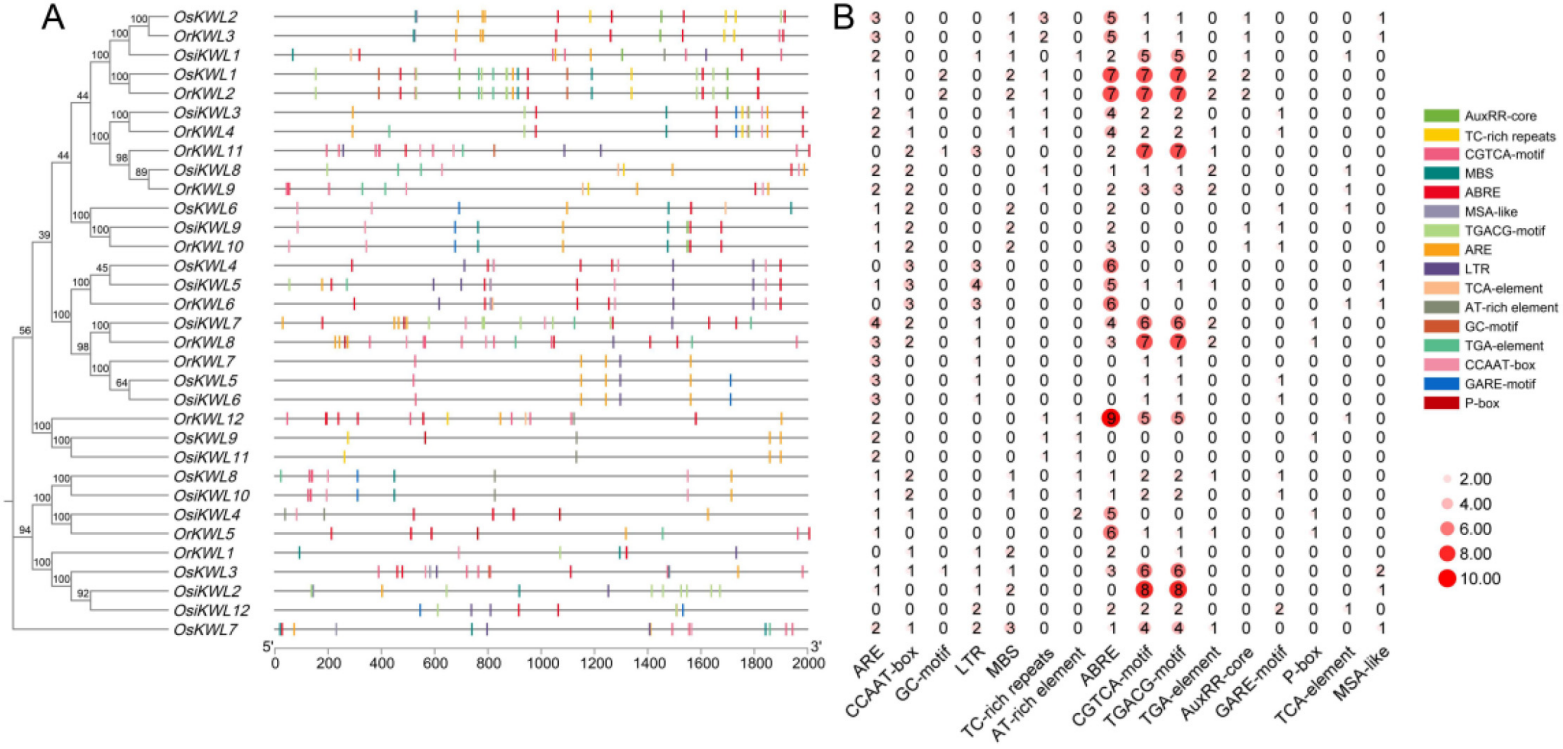
Visualization analysis of cis-acting elements in the promoter region of the *KWL* gene in three *Oryza* species. **(A)** Cis-acting elements were identified in the 2.0 kb upstream promoter regions of *KWL* genes. All depicted elements are located on the positive strand. **(B)** The numbers in the heatmap indicate the abundance of the corresponding cis-acting elements.

To explore the potential biological functions of *KWL* genes in rice, we extracted the 2000 bp upstream sequences of *KWL* genes and predicted cis-acting elements in the promoter regions using the PlantCARE database (**Figure 5**). A variety of cis-acting elements were identified, particularly those associated with biotic/abiotic stress responses and phytohormone signaling. Notably, cis-acting elements involved in abscisic acid responsiveness (ABRE) and methyl jasmonate responsiveness (CGTCA-motif and TGACG-motif) were significantly enriched. Additionally, a small number of elements responsive to salicylic acid, gibberellin, and auxin were detected. The ARE (anaerobic response element), essential for the anaerobic induction, was also widely distributed in the promoter regions of *KWL* genes. These results suggest that *KWL* genes may play critical roles in stress adaptation and phytohormone signaling in rice.

### 3.8 Tissue-specific expression profiles of *OsKWL* genes

**Figure 6:**
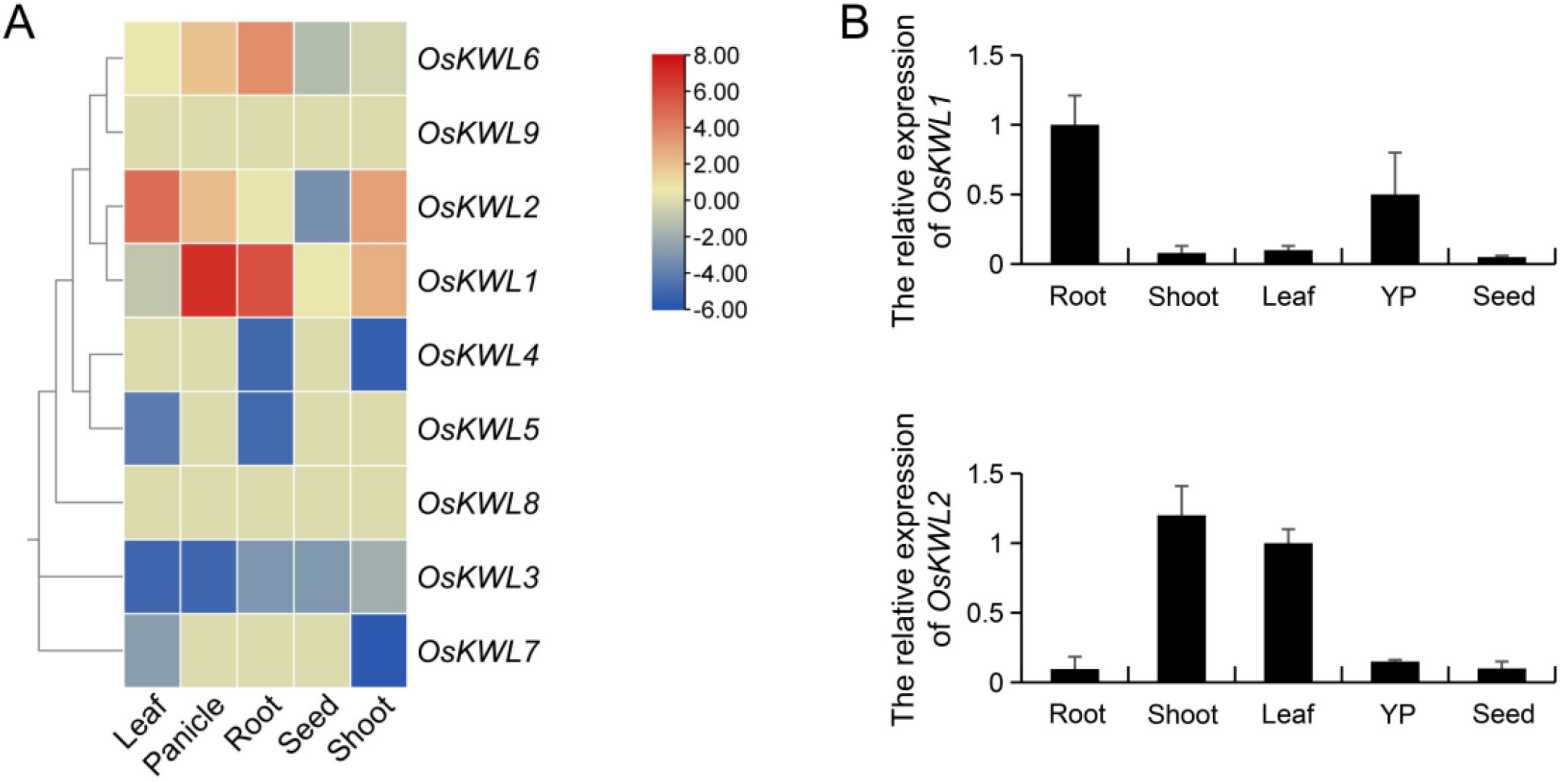
Expression pattern of *OsKWL* genes. **(A)** Expression analysis of the *OsKWLs* in different tissues. The heatmap was created based on the log2(FPKM) value of *OsKWLs*. Differences in gene expression are colored, with red for high expression and blue for low expression. **(B)** qRT-PCR analysis of *OsKWL1* and *OsKWL2* in different tissues, including the root, shoot, leaf, young panicle (YP) and seed. *OsActin* was used as the control, and all values are means ± SD of three biological replicates.

Tissue-specific patterns of gene expression offer essential insights into functional contributions and regulatory networks within distinct plant tissues. To investigate the tissue-specific expression patterns of *OsKWL* genes, we analyzed transcriptome data obtained from the RED database. The resulting heatmap revealed significantly divergent expression patterns among *OsKWL* members (**Figure 6A**). Notably, *OsKWL1* showed relatively high expression in panicles and roots but was nearly undetectable in leaves. In contrast, *OsKWL2* displayed the highest expression in leaves and shoots with almost no expression in roots. The remaining members showed generally low expression across all tissues examined. This preliminary analysis identified *OsKWL1* and *OsKWL2* as primary candidate genes for further functional investigation due to their strong and tissue-specific expression patterns.

To validate these bioinformatic predictions and quantify expression levels more precisely, we performed qRT-PCR analysis of the two candidate genes, *OsKWL1* and *OsKWL2*, across the same panel of tissues (**Figure 6B**). Consistent with the heatmap results, *OsKWL1* expression was significantly enriched in roots and substantially lower in aerial tissues. Similarly, *OsKWL2* expression was confirmed to be highest in shoots.

### 3.9 Hormone response analysis of *OsKWL* genes

**Figure 7:**
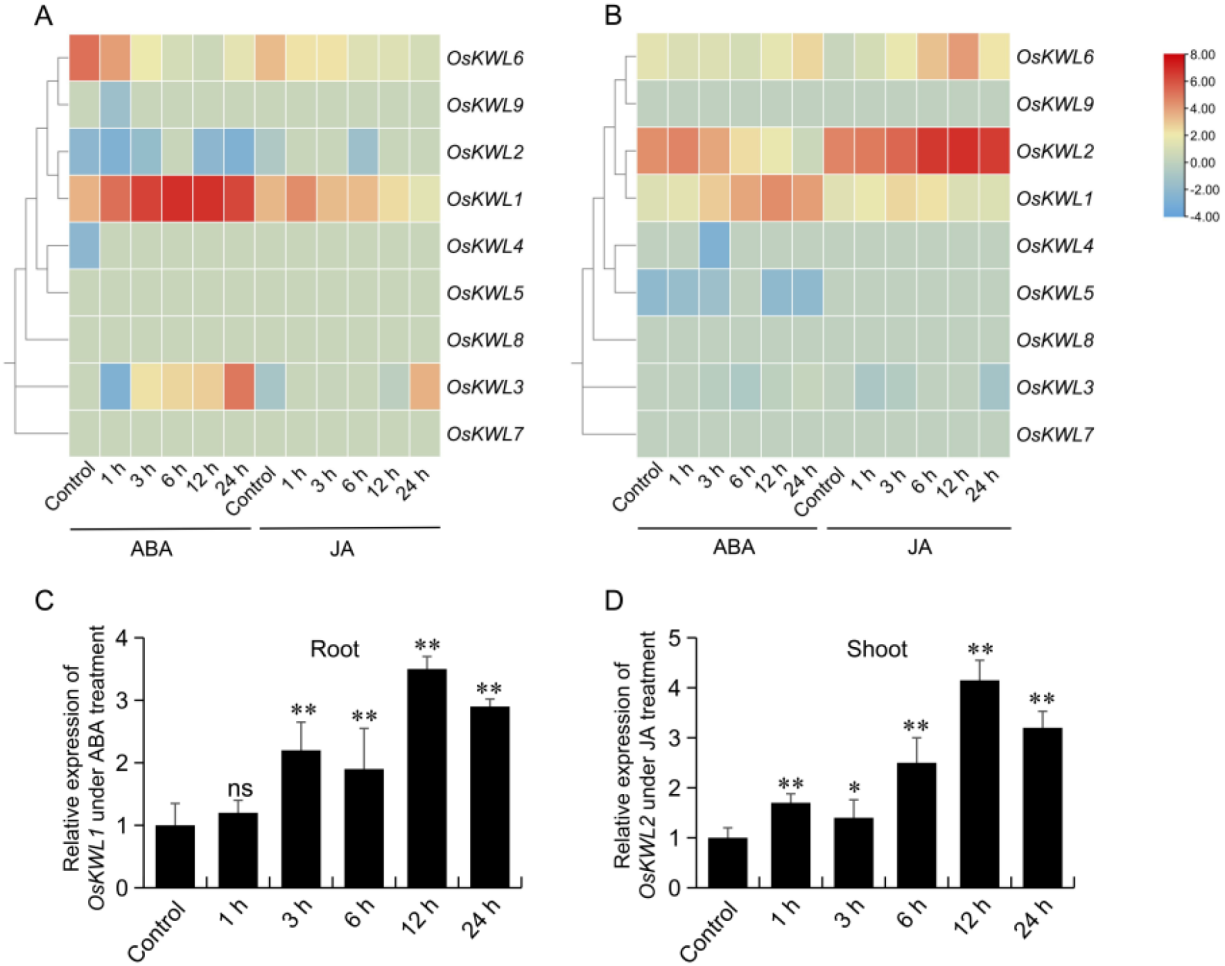
Expression analysis of *OsKWL* genes under hormone treatments. **(A, B)** Expression profiles of *OsKWLs* in roots **(A)** and shoots **(B)** following ABA and JA treatments. Data were obtained from the Rice RNA-seq Database. **(C, D)** Expression analysis of *OsKWL1* in roots after ABA treatment and *OsKWL2* in shoots after JA treatment was performed using qRT-PCR, with *OsActin* as the reference gene for normalization. All values are means ± SD of three biological replicates. ns, no significant differences; *P < 0.05; **P < 0.01 by Student’s *t*-test.

Promoter element analysis revealed that the promoter regions of *OsKWL* genes were significantly enriched in cis-acting elements such as ABRE, CGTCA-motif, and TGACG-motif, which are associated with ABA and JA responsiveness. To further investigate the response patterns of *OsKWL* members to exogenous ABA and JA, we analyzed public transcriptome data. The results showed that in roots, *OsKWL1* exhibited strong responsiveness to ABA treatment, whereas in shoots, exogenous JA significantly induced the expression of *OsKWL2* (**Figures 7A, B**). By contrast, other *OsKWL* members did not display marked responses to the hormone treatments. These findings suggest that *OsKWL1* and *OsKWL2* may be involved in ABA and JA -mediated stress responses, respectively.

To further validate these bioinformatics-based predictions, two-week-old Nipponbare seedlings were treated with exogenous ABA and JA, and the expression patterns of the target genes were quantified using qRT-PCR. The results demonstrated that the expression of *OsKWL1* in roots was significantly induced by ABA, peaking at 12 h after treatment, with a relative expression level approximately 3.5-fold higher than that of the control (**Figure 7C**). Similarly, JA treatment markedly upregulated *OsKWL2* expression in shoots, which also peaked at 12 h, reaching about 4.2-fold that of the control (**Figure 7D**).

### 3.10 Subcellular localization analysis

**Figure 8:**
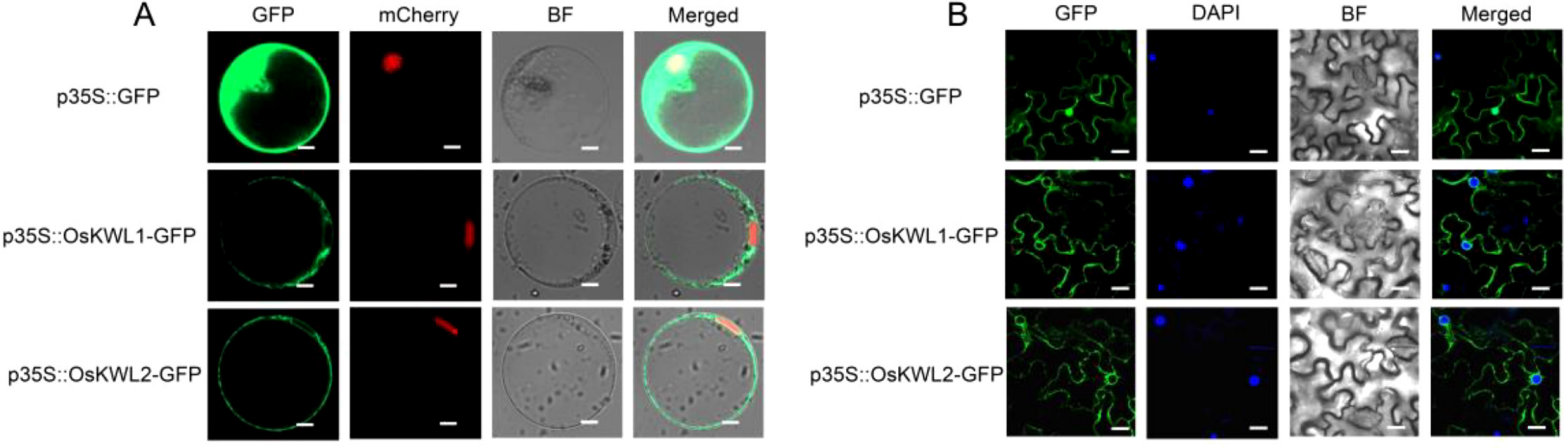
Subcellular localization of OsKWL1 and OsKWL2. **(A)** Subcellular localization of OsKWL1 and OsKWL2 observed in rice protoplasts. The DWARF 53-mCherry fusion protein serves as a nuclear localization marker. Scale bar, 5 μm. **(B)** Subcellular localization of OsKWL1 and OsKWL2 observed in *N. benthamiana* leaves. Scale bar, 20 μm.

To investigate the subcellular localization of OsKWL1 and OsKWL2 proteins, we generated a green fluorescent protein (GFP) fusion construct (OsKWL1-GFP and OsKWL2-GFP) under the control of 35S promoter. The recombinant constructs were first transiently expressed in rice protoplasts. Confocal fluorescence microscopy revealed that the green fluorescence of the OsKWL1::GFP and OsKWL2::GFP fusion proteins was widely distributed throughout the cytoplasm and showed no co-localization with the nuclear marker (**Figure 8A**). To further examine whether similar localization patterns could be observed in another transient expression system, OsKWL1-GFP and OsKWL2-GFP fusion constructs were further expressed in *N. benthamiana* leaves via *Agrobacterium*-mediated transformation. Consistent with our observations in rice protoplasts, the fluorescence signal was predominantly localized to the cytoplasm (**Figure 8B**). Together, these results from two independent transient expression assays suggest that OsKWL1 and OsKWL2 perform their biological functions in the cytoplasm.

### 3.11 Autoactivation activity of OsKWL1 and OsKWL2 in yeast

**Figure 9:**
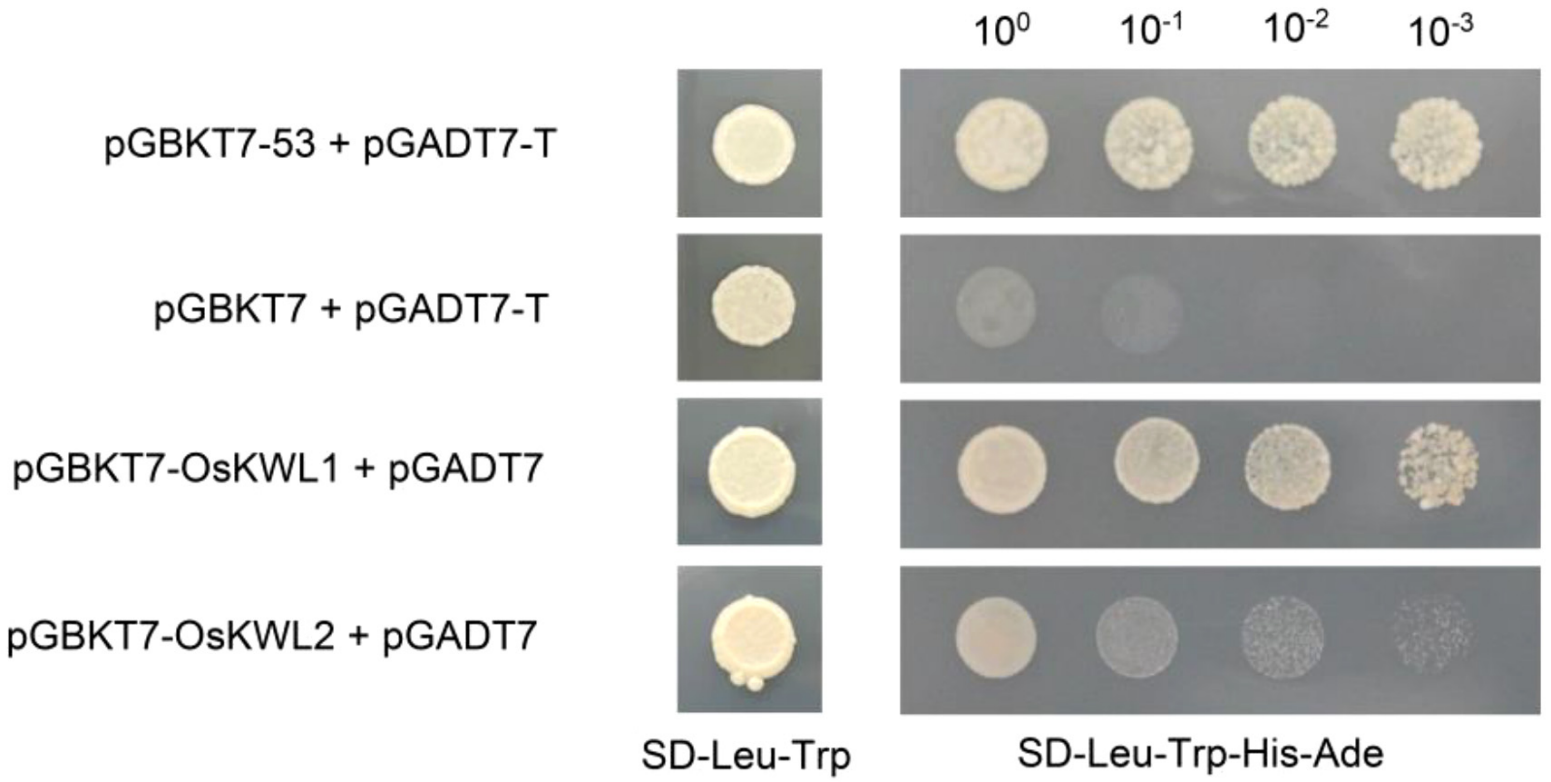
Transcription autoactivation of OsKWL1 and OsKWL2 in yeast. The full-length coding sequences of *OsKWL1* and *OsKWL2* were cloned into the pGBKT7 vector. The combinations pGBKT7-53 + pGADT7-T and pGBKT7 + pGADT7-T were used as positive and negative controls, respectively.

To further investigate the molecular functions of *OsKWL1* and *OsKWL2* and to provide a basis for subsequent screening of their interacting proteins using the yeast two-hybrid system, we assessed the autoactivation activity of both proteins in yeast (**Figure 9**). The results showed that yeast strain Y2HGold transformed with the pGBKT7-OsKWL1 vector grew normally on SD/–Leu–Trp/–His/–Ade selective medium, similar to the positive control (pGBKT7-53), indicating that OsKWL1 possesses strong autoactivation activity. In contrast, yeast harboring the empty pGBKT7 vector (negative control) failed to grow under the same selective media, confirming the absence of autoactivation. Notably, OsKWL2 exhibited only weak autoactivation activity.

## 4 Discussion

The *KWL* gene family, while implicated in plant stress responses, remains one of the least functionally characterized gene families, particularly in monocot crops such as rice. Its absence in the model Brassicaceae family further underscores the need for functional investigations in crops. Our comprehensive genome-wide analysis of *KWL* genes in three *Oryza* species not only provides a foundational catalog of its members but, more importantly, unveils critical structural and expression features that point towards their putative functional roles in rice development and environmental adaptation.

We identified a total of 9, 12, and 12 *KWL* genes in *Os*, *Osi*, and *Or* genomes, respectively (**Figure 3**). The number of KWL genes in these rice species is comparable to that reported in tomato (Su et al., 2024). Analysis of physicochemical properties revealed a high degree of similarity among most *KWL* family members (**Supplementary Table S1**). Furthermore, phylogenetic analysis showed that the *KWL* members from the three *Oryza* species form distinct clusters, clearly separated from those of maize and tomato (**Figure 1**). This indicates that the functions of these genes are relatively conserved within *Oryza* species and may be associated with rice specific biological processes.

Similar gene structures and conserved motifs often indicate related biological functions (Kaczanowski and Zielenkiewicz, 2010). Consistent with this principle, our analysis revealed that KWL proteins within the same phylogenetic clade share similar conserved motifs, suggesting they may perform similar biological roles (**Figure 2**). The striking prevalence of intronless genes within the rice KWL family represents a notable structural feature with potential functional significance. This genomic architecture, also observed in other rapidly induced stress-responsive genes, potentially facilitates swift transcript processing and protein synthesis in response to environmental cues. This observation aligns well with the abundance of stress- and hormone-related cis-elements in their promoters and supports the hypothesis that *KWL* genes primarily function in mediating rapid adaptive responses in rice.

Members of the *KWL* gene family are primarily distributed across chromosomes 2, 4, 6, and 10. Notably, in all three species, the majority of members formed tight clusters on chromosome 10 (**Figure 3**). Similar clustering patterns have also been reported for several disease-resistance gene families in rice, such as NBS-LRR and RLK, which may enhance resistance efficiency through coordinated regulation (Long et al., 2024; Vij et al., 2008; Zhou et al., 2004). Gene duplication events, including tandem, segmental, and whole-genome duplication, play critical roles in adaptive evolution and species diversification by driving functional divergence, enhancing genomic plasticity, and increasing the complexity of regulatory networks (Clark and Donoghue, 2018). Interestingly, no tandem or segmental duplication events were detected among the *KWL* members in *Os*, *Osi*, or *Or*. This suggests that the expansion of this family may have occurred through alternative mechanisms, such as retrotransposon-mediated replication. Comparative synteny analysis revealed that *Os* shares four and three pairs of homologous *KWL* genes with *Osi* and *Or*, respectively (**Figures 4A, B**). Furthermore, synteny analysis indicated a closer evolutionary relationship between rice and other monocot species like maize and sorghum (**Figures 4C, D**; **Supplementary Figure S4**), supporting the hypothesis that monocots diverged relatively recently and exhibit stronger genomic conservation.

Cis-acting elements in gene promoter regions play essential roles in regulating spatiotemporal expression, environmental responses, and developmental processes (Hernandez-Garcia and Finer, 2014; Cui et al., 2023; Marand et al., 2023). Promoter analysis of the *KWL* genes revealed an abundance of cis-acting elements related to hormone and stress responses (**Figure 5**). A significant enrichment of ABA and JA responsive elements (ABRE, CGTCA-motif, TGACG-motif) were observed, strongly suggesting that this gene family may be extensively involved in the regulation of ABA and JA signaling pathways. Our expression profiling identified *OsKWL1* and *OsKWL2* as the primary functional candidates, exhibiting strong, tissue-specific responses to hormones (**Figure 7**). The specific induction of *OsKWL1* in roots by ABA strongly suggests its role in root-specific ABA signaling pathways, potentially contributing to drought tolerance or other soil-related stresses. Conversely, the shoot-specific induction of *OsKWL2* by JA positions it as a likely mediator of JA-dependent defense responses against herbivores or pathogens in aerial tissues. This spatial and hormonal segregation indicates a sophisticated division of labor within the *KWL* family that coordinates whole-plant stress resilience. The generally low expression of other members suggests they may serve as redundant backups or be activated under specific, unexamined conditions, such as particular developmental stages or specialized biotic interactions. It should be noted that the expression analyses presented here are currently limited to the *japonica* subspecies (Nipponbare). While our promoter analysis predicts conserved regulatory roles across rice subspecies, future investigations incorporating expression profiling in *indica* cultivars (such as Minghui 63) will be crucial to validate the conservation of these expression patterns and to explore potential subspecies-specific regulatory differences.

Although previous studies in other plant species have linked KWL proteins to defense-related processes, our database survey revealed no significant induction of rice *KWL* genes under pathogen infection conditions. This observation suggests that *KWL* genes in rice may not be directly activated by pathogen challenge, or that their defense-related functions are conditional or tissue-specific. It is also possible that *KWL* genes participate in disease resistance through indirect mechanisms, such as cross-talk with hormone signaling pathways.

One of the most intriguing findings of our study is the apparent paradox between the subcellular localization and transactivation activity of OsKWL1 and OsKWL2. While both proteins were consistently localized to the cytoplasm in our transient assays (**Figure 8**), OsKWL1 exhibited strong autoactivation activity in the yeast nucleus (**Figure 9**). This apparent discrepancy suggests that OsKWL1 and OsKWL2 may not be statically localized in the cytoplasm but could undergo stimulus-dependent nucleocytoplasmic shuttling—a regulatory mechanism widely observed in plants. For instance, the glycolytic enzyme GAPC translocates from the cytoplasm to the nucleus under heat stress in *Arabidopsis thaliana* (Kim et al., 2020), and the plasma membrane protein CRPK1 mediates nuclear import of 14-3–3 proteins in response to cold stress (Liu et al., 2017). Additionally, the transcription factor BZR1 is dephosphorylated and accumulates in the nucleus upon brassinosteroid (BR) treatment in *Arabidopsis* (Wang et al., 2021). Thus, we hypothesize that OsKWL1 (and potentially OsKWL2) is predominantly cytoplasmic under normal conditions but may translocate into the nucleus upon specific stimuli, such as post-translational modifications (e.g., phosphorylation), interaction with binding partners, or under specific stress conditions that trigger JA/ABA signaling. This conditional nuclear import would allow it to function as a transcriptional regulator, potentially activating a suite of downstream defense-related genes. This model positions OsKWL1 not merely as a passive protein but as a dynamic signaling node, whose regulatory function is tightly controlled at the level of cellular compartmentalization. Future work, such as identifying their interaction partners and monitoring localization under hormone treatment, will be crucial to validate this hypothesis.

## Data Availability

The original contributions presented in the study are included in the article/**Supplementary Material**. Further inquiries can be directed to the corresponding author.
