# Supplementary material for: Genome-wide identification and functional characterization of the KWL gene family in three Oryza species
Source: Front Plant Sci. 2025 Nov 19;16:1707474. doi: 10.3389/fpls.2025.1707474 (PMC12672553; doi:10.3389/fpls.2025.1707474)
Supplement: Supplementary file 1 [file DataSheet1.docx]

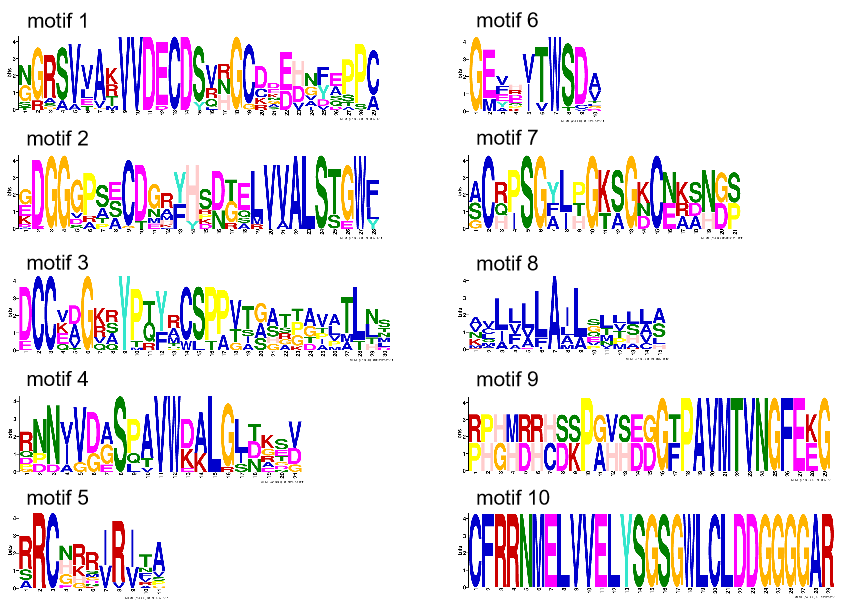


**Supplementary Figure S1.** Conserved motifs of 33 *KWLs*.





**Supplementary Figure S2.** The tertiary structure of proteins of the KWL proteins.


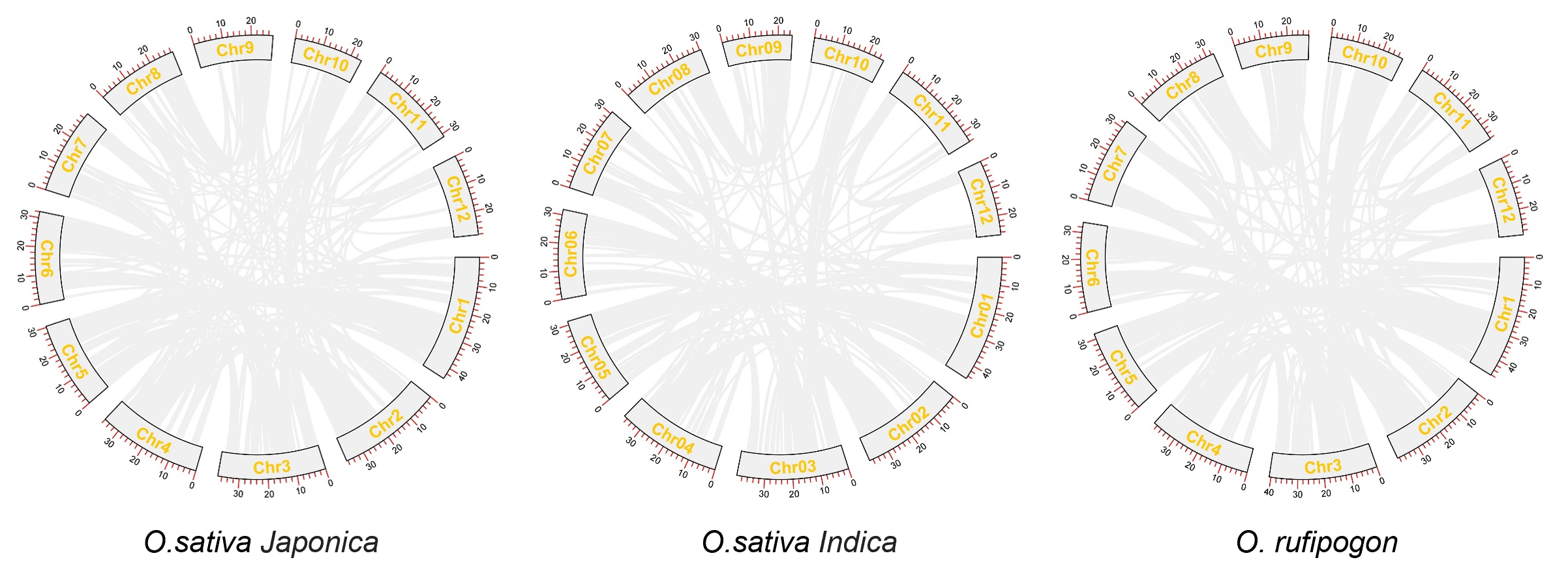


**Supplementary Figure S3.** No collinear gene pairs were detected in any of the three rice species.

**
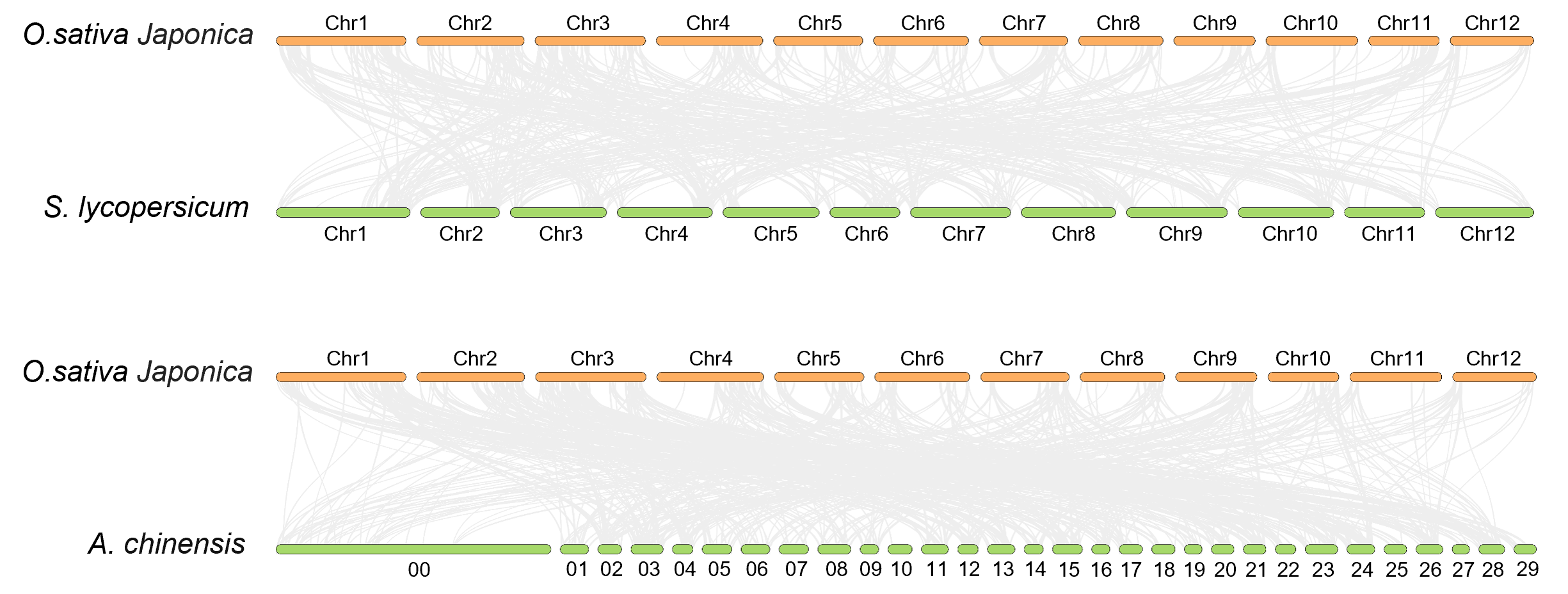
**

**Supplementary Figure S4.** No collinear gene pairs were identified between *KWL* genes in rice and dicots (tomato and kiwifruit).

**Supplementary Table S1.** Identification information of *KWLs* in three rice species.

|  | **ID** | **Number of Amino Acid (AA)** | **Molecular Weight (Da)** | **Theoretical pI** | **Instability Index** | **Aliphatic Index** | **Grand Average of Hydropathicity** | **Subcellular Localization** |
| --- | --- | --- | --- | --- | --- | --- | --- | --- |
| *Oryza sativa* ssp. *japonica* | *OsKWL1* | 192 | 20395.74 | 6.07 | 55.33 | 68.65 | -0.406 | Nucleus |
|  | *OsKWL2* | 183 | 19096.04 | 4.36 | 48.45 | 65.57 | -0.265 | Cell membrane. Nucleus |
|  | *OsKWL3* | 253 | 27630.79 | 9.64 | 42.29 | 74.70 | -0.146 | Cell membrane. Cell wall. Chloroplast. Nucleus |
|  | *OsKWL4* | 167 | 17237.24 | 5.67 | 55.81 | 70.72 | -0.193 | Chloroplast. Nucleus. Peroxisome |
|  | *OsKWL5* | 162 | 17194.18 | 6.24 | 64.85 | 72.84 | -0.319 | Chloroplast. Nucleus. Peroxisome |
|  | *OsKWL6* | 213 | 21602.89 | 4.42 | 67.08 | 70.19 | -0.063 | Nucleus |
|  | *OsKWL7* | 1202 | 139015.14 | 7.54 | 55.26 | 65.86 | -0.855 | Nucleus |
|  | *OsKWL8* | 196 | 20869.74 | 10.21 | 65.98 | 68.72 | -0.282 | Nucleus |
|  | *OsKWL9* | 216 | 22175.97 | 8.07 | 43.38 | 69.95 | -0.192 | Cytoplasm. Nucleus. Peroxisome |
| *Oryza sativa* ssp. *indica* | *OsiKWL1* | 181 | 19130.23 | 4.53 | 47.08 | 67.4 | -0.292 | Cell wall |
|  | *OsiKWL2* | 248 | 26653.44 | 9.03 | 49.65 | 73.1 | -0.142 | Cell wall. Chloroplast. Nucleus |
|  | *OsiKWL3* | 195 | 20239.47 | 4.92 | 43.75 | 66.1 | -0.257 | Nucleus |
|  | *OsiKWL4* | 179 | 19110.75 | 7.62 | 51.68 | 85.53 | 0.049 | Cell membrane. Chloroplast. Nucleus |
|  | *OsiKWL5* | 167 | 17237.24 | 5.67 | 55.81 | 70.72 | -0.193 | Chloroplast. Nucleus. Peroxisome |
|  | *OsiKWL6* | 162 | 17175.14 | 6.15 | 61.32 | 72.84 | -0.311 | Chloroplast. Cytoplasm. Nucleus |
|  | *OsiKWL7* | 132 | 13862.76 | 6.72 | 46.52 | 85 | -0.117 | Peroxisome |
|  | *OsiKWL8* | 239 | 24939.13 | 6.37 | 37.09 | 69.92 | -0.236 | Cell membrane. Chloroplast. Nucleus. Peroxisome. |
|  | *OsiKWL9* | 213 | 21602.83 | 4.42 | 64.58 | 70.19 | -0.077 | Nucleus |
|  | *OsiKWL10* | 212 | 22688.86 | 10.47 | 67.12 | 69.95 | -0.266 | Nucleus |
|  | *OsiKWL11* | 213 | 22043.89 | 8.36 | 44.7 | 71.41 | -0.195 | Cell wall. Chloroplast. Cytoplasm. Nucleus. Peroxisome. |
|  | *OsiKWL12* | 246 | 26496.18 | 9.02 | 54.25 | 68.94 | -0.195 | Nucleus |
| *Oryza rufipogon* | *OrKWL1* | 182 | 19834.42 | 7.54 | 64.98 | 66.48 | -0.521 | Nucleus |
|  | *OrKWL2* | 192 | 20395.74 | 6.07 | 55.33 | 68.65 | -0.406 | Nucleus |
|  | *OrKWL3* | 181 | 18893.77 | 4.36 | 48.2 | 65.75 | -0.288 | Cell membrane. Cell wall. Nucleus |
|  | *OrKWL4* | 195 | 20239.47 | 4.92 | 43.75 | 66.1 | -0.257 | Nucleus |
|  | *OrKWL5* | 97 | 10445.93 | 9.24 | 49.74 | 84.33 | -0.039 | Chloroplast. Nucleus |
|  | *OrKWL6* | 167 | 17221.28 | 5.91 | 55.81 | 72.46 | -0.147 | Chloroplast. Nucleus. Peroxisome. |
|  | *OrKWL7* | 162 | 17148.09 | 6.24 | 60.95 | 72.84 | -0.337 | Chloroplast. Cytoplasm. Nucleus |
|  | *OrKWL8* | 74 | 7893.8 | 5.2 | 47.57 | 72.43 | -0.243 | Nucleus |
|  | *OrKWL9* | 190 | 19639.78 | 4.79 | 40.42 | 66.32 | -0.221 | Cell membrane. Chloroplast. Nucleus |
|  | *OrKWL10* | 213 | 21602.83 | 4.42 | 64.58 | 70.19 | -0.077 | Nucleus |
|  | *OrKWL11* | 307 | 32262.07 | 5.65 | 48.9 | 69.61 | -0.252 | Cell wall. Chloroplast. Nucleus. Peroxisome |
|  | *OrKWL12* | 218 | 22383.29 | 8.57 | 46.05 | 71.56 | -0.17 | Chloroplast. Nucleus. Peroxisome |

**Supplementary Table S2.** Homologous gene Ka/Ks values.

| Gene pair | Ka | Ks | Ka/Ks |
| --- | --- | --- | --- |
| *OsKWL2/OsiKWL1* | 0.035345202 | 0.030345479 | 1.164760067 |
| *OsKWL3/OsiKWL2* | 0.052328743 | 0.096478883 | 0.542385455 |
| *OsKWL4/OsiKWL5* | 0 | 0.0075854 | 0 |
| *OsKWL8/OsiKWL10* | 0.034442243 | 0.05803697 | 0.593453512 |
| *OsKWL1/OrKWL2* | 0 | 0 | NaN |
| *OsKWL2/OrKWL3* | 0 | 0 | NaN |
| *OsKWL4/OrKWL6* | 0 | 0 | NaN |
| *OsKWL1/GRMZM2G073114* | 0.42352 | 0.734251 | 0.576806 |
| *OsKWL5/GRMZM2G165149* | 0.243318 | 0.892627 | 0.272586 |
| *OsKWL6/GRMZM2G418833* | 0.236282 | 0.531525 | 0.444537 |
| *OsKWL6/GRMZM2G073114* | 0.250453 | 0.534141 | 0.46889 |
| *OsKWL1/EES07240* | 0.225356 | 0.392436 | 0.57425 |
| *OsKWL4/OQU91589* | 0.488812 | 0.989825 | 0.493837 |
| *OsKWL5/OQU91588* | 0.51968 | 0.954888 | 0.544232 |
| *OsKWL8/OQU91584* | 0.589593 | 1.790126 | 0.329358 |

**Supplementary Table S3.** Primers used in this study

| Purpose | Primer name | Primer sequence (5′ – 3′) |
| --- | --- | --- |
| Subcellular localization | OsKWL1-PAN580-CF | TCTTAAGTCCGGAGCTAGCTCTAGAATGGCGACGACGAATTGCCT |
|  | OsKWL1-PAN580-CR | CCTCGCCCTTGCTCACCATGGATCCTGCGTCCGACCAAGTGATAT |
|  | OsKWL2-PAN580-CF | TCTTAAGTCCGGAGCTAGCTCTAGAATGGCGATGACCAATTGCCT |
|  | OsKWL2-PAN580-CR | CCTCGCCCTTGCTCACCATGGATCCTGCGTCCGACCAAGTGATAT |
|  | OsKWL1-1300-CF | GGGACTCTTGACGAGCTCGGTACCATGGCGACGACGAATTGCCT |
|  | OsKWL1-1300-CR | CCTTGCTCACCATGTCGACTCTAGATGCGTCCGACCAAGTGATAT |
|  | OsKWL2-1300-CF | GGGACTCTTGACGAGCTCGGTACCATGGCGATGACCAATTGCCT |
|  | OsKWL2-1300-CR | CCTTGCTCACCATGTCGACTCTAGATGCGTCCGACCAAGTGATAT |
| autoactivation activity | OsKWL1-BD-CF | gcatatggccatggaggccgaattcATGGCGACGACGAATTGCCT |
|  | OsKWL1-BD-CR | GCGGCCGCTGCAGGTCGACGGATCCTGCGTCCGACCAAGTGATAT |
|  | OsKWL2-BD-CF | gcatatggccatggaggccgaattcATGGCGATGACCAATTGCCT |
|  | OsKWL2-BD-CR | GCGGCCGCTGCAGGTCGACGGATCCTGCGTCCGACCAAGTGATAT |
| qRT-PCR | Actin-qPCR-F | TTCCTACATCGCCCTGGACT |
|  | Actin-qPCR-R | AGCCTTGGCAATCCACATCT |
|  | OsKWL1-qPCR-F | GTCCGTGATACTGTTCCAAATCC |
|  | OsKWL1-qPCR-R | TTCGGATGTGACCGTACTCG |
|  | OsKWL2-qPCR-F | TCCTACCCGACGTACACTTG |
|  | OsKWL2-qPCR-R | TGTGGTACATCTCGTCGCAC |
